# Does Stress Increase the Risk of Atopic Dermatitis in Adolescents? Results of the Korea Youth Risk Behavior Web-Based Survey (KYRBWS-VI)

**DOI:** 10.1371/journal.pone.0067890

**Published:** 2013-08-05

**Authors:** Jeoung A. Kwon, Eun-Cheol Park, Minjee Lee, Ki-Bong Yoo, Sohee Park

**Affiliations:** 1 Department of Public Health, Graduate School, Yonsei University, Seoul, Republic of Korea; 2 Institute of Health Services Research, Yonsei University College of Medicine, Seoul, Republic of Korea; 3 Department of Preventive Medicine, Yonsei University College of Medicine, Seoul, Republic of Korea; 4 Department of Biostatistics, Graduate School of Public Health, Yonsei University, Seoul, Republic of Korea; Gentofte University Hospital, Denmark

## Abstract

This study investigated the relationship between level of stress in middle and high school students aged 12–18 and risk of atopic dermatitis. Data from the Sixth Korea Youth Risk Behavior Web-based Survey (KYRBWS-VI), a cross-sectional study among 74,980 students in 800 middle schools and high schools with a response rate of 97.7%, were analyzed. Ordinal logistic regression analyses were conducted to determine the relationship between stress and atopic dermatitis with severity. A total of 5,550 boys and 6,964 girls reported having been diagnosed with atopic dermatitis. Younger students were more likely to have atopic dermatitis. Interestingly, the educational level of parents was found to be associated with having atopic dermatitis and having more severe condition. In particular, girls with mothers with at least college education had a 41% higher risk of having atopic dermatitis and severe atopic condition (odds ratio (OR)) = 1.41, 95% CI, 1.22–1.63; *P*<0.0001) compared with those with mothers who had attended middle school at most. Similar trend was shown among both boys and girls for their father's education level. The stress level was found to be significantly associated with the risk of atopic dermatitis. Compared to boys with who reported “no stress”, boys with “very high” stress had 46% higher the risk of having more severe atopic dermatitis (OR = 1.46, 95% CI, 1.20–1.78; *P*<0.0001), 44% higher (OR = 1.44, 95% CI, 1.19–1.73; *P*<0.0001) with “high” stress, and 21% higher (OR = 1.21, 95% CI, 1.00–1.45; *P* = 0.05) with “moderate” stress. In contrast, we found no statistically significant relationship between stress and atopic dermatitis in girls. This study suggests that stress and parents' education level were associated with atopic dermatitis. Specifically, degree of stress is positively correlated with likelihood of being diagnosed with this condition and increasing the severity.

## Introduction

According to the Korea Youth Risk Behavior Web-based Survey (KYRBWS), the percentage of middle and high school students diagnosed with atopic dermatitis gradually increased in Korea from 2007 to 2011, with incidences of 16.8% in 2007, 18.2% in 2008, 18.6% in 2009, 21.7% in 2010, and 23.0% in 2011. For this reason, atopic dermatitis should be considered a significant condition among Korean adolescents.

Atopic dermatitis is a chronic inflammatory skin disorder characterized by eczematous lesions and pruritus [1], [2]. The typical characteristics of this condition are chronic, recurrent pruritus and lichenification at typical sites on the body, and atopic comorbidities include asthma, rhinitis, and conjunctivitis [3]. Atopic dermatitis may be caused by genetic predisposition and environmental conditions [1], including hereditary factors, allergens, and neurogenous and immunological factors [4], [5]. However, the major etiologic agent remains unknown [1]. Atopic dermatitis may cause psychosocial problems such as anxiety, depression, sleep disorders, emotional excitability, stigmatization, social isolation, and discrimination [6]–[12]. Of the many factors related to atopic dermatitis, psychological stress is considered to be among the most important [13]. Additionally, psychological stress and symptoms of atopic dermatitis seem to contribute to a vicious cycle [14].

Several studies have demonstrated a relationship between stress and atopic dermatitis [15]–[23]. Hashizume et al. reported that psychological stress exacerbates atopic dermatitis [14]. Additionally, several studies have shown that, compared with healthy controls, patients with atopic dermatitis react differently to laboratory stress and experience a worsening of the skin after exposure to stress [24]–[27]. Using the Trier Social Stress Test (TSST), Buske-Kirschbaum et al. compared atopic leukocyte subsets, serum immunoglobulin E (IgE) levels, and cytokine concentrations in patients with dermatitis and non-atopic controls who were stressed in front of an audience. Although no difference in the numbers of serum lymphocytes, monocytes, neutrophils, and basophils was detected between the two groups, the eosinophil and IgE levels were significantly higher in patients with atopic dermatitis compared with non-atopic controls [28]. Moreover, production of cortisol and adrenocorticotropic hormone was decreased in patients with atopic dermatitis compared with non-atopic controls after experimental TSST stressors [28]. The results of these studies show that the immunological reactions of patients with atopic dermatitis differ from those of non-atopic individuals subjected to psychological stressors [1].

The response of the stress-related hypothalamic–pituitary–adrenal (HPA) axis to psychological stressors differs by age and sex in humans [29]. Some psychological stress studies have shown that young men have stronger cortisol responses than do young women after acute real-life psychological stress [29].

The majority of studies comparing individuals with atopic dermatitis with controls have targeted adult populations. Although several studies have investigated atopic dermatitis in adolescents, there are no studies analyzing Asian populations or the association between degree of stress and atopic dermatitis. Adolescents in Korea are at high risk for stress because of the highly competitive atmosphere at school. Therefore, this study targeted middle and high school boys and girls aged 12–18 to investigate the relationship between stress and risk of atopic dermatitis adjusting for age, current smoking status, current alcohol consumption, maternal educational level, paternal educational level, economic status, living situation, and type of city. A particular focus of this study was the association between level of stress and atopic dermatitis.

## Materials and Methods

### Subjects

The Sixth Korea Youth Risk Behavior Web-based Survey (KYRBWS -VI) survey was conducted in 2010 by the Korea Centers for Disease Control and Prevention (KCDCP). The complex research design included multistage sampling, stratification, and clustering. The purpose of this survey was to determine the prevalence of health-risk behaviors in Korean adolescent students in the seventh to the twelfth grades. All data collection procedures have been reported by the KCDCP [30]. The reliability and validity of the data collected using the questionnaire have been assessed by several studies [31], [32]. The nationwide KYRBWS-VI survey data are maintained by KCDCP and are publicly available through the KCDCP website. We submitted a data request form on the KCDCP website, and after the internal review by KCDCP and approval, we obtained the KYRBWS-VI survey data with all private information anonymously dealt with.

During the survey, participants were assigned identification (ID) numbers and guaranteed anonymity. Teachers randomly assigned a computer to each student and provided the survey information. After the process was sufficiently explained, each student accessed the website using his or her ID number and completed the survey. In 2010, 74,980 participants from 800 schools (400 middle schools and 400 high schools) participated. Among 74,980 participants, 73,238 students responded to the survey. The response rate was 97.7%. From 73,238 responders, 17,507 students were excluded due to missing information on relevant covariates, leaving a total of 55,731 (28,501 boys and 27,230 girls) for the cross-sectional analysis. Among 55,731 participants, 12,514 participants were diagnosed with atopic dermatitis. Because the KYRBWS-VI is a secondary data that do not contain private information, our study did not have to address ethical concerns. Table 1 shows the characteristics of the participants.

**Table 1:** General characteristics of study participants.

Variables	Boys (n = 28,501)	Girls (*n* = 27,230)	Total (*n* = 55,731 )	P-value
**Age (years)**	*n* (%)	*n* (%)	*n* (%)	<0.0001
12	1,402 (4.9)	1,460 (5.4)	2,862 (5.1)	
13	4,494 (15.8)	4,155 (15.3)	8,649 (15.5)	
14	4,714 (16.5)	4,418 (16.2)	9,132 (16.4)	
15	4,954 (17.4)	4,658 (17.1)	9,612 (17.3)	
16	5,070 (17.8)	4,878 (17.9)	9,948 (17.9)	
17	5,020 (17.6)	4,967 (18.2)	9,987 (17.9)	
18	2,847 (10.0)	2,694 (9.9)	5,541 (9.9)	
**Current smoking**				<0.0001
Nonsmoking	2,3807 (83.5)	25,514 (93.7)	49,321 (88.5)	
Smoking	4,694 (16.5)	1,716 (6.3)	6,410 (11.5)	
**Current alcohol consumption**				<0.0001
Nondrinking	21,633 (75.9)	22,176 (81.4)	43,809 (78.6)	
Drinking	6,868 (24.1)	5,054 (18.6)	11,922 (21.4)	
**Educational level of mother**				<0.0001
Middle school or lower	1,927 (6.8)	1,859 (6.8)	3,786 (6.8)	
High school	15,699 (55.1)	15,765 (57.9)	31,464 (56.5)	
College or higher	10,875 (38.2)	9,606 (35.3)	20,481 (36.8)	
**Educational level of father**				<0.0001
Middle school or lower	1,988 (7.0)	1,796 (6.6)	3,784 (6.8)	
High school	12,028 (42.2)	11,930 (43.8)	23,958 (43.0)	
College or higher	14,485 (50.8)	13,504 (49.6)	27,989 (50.2)	
**Economic status**				<0.0001
Very wealthy	2,529 (8.9)	1,309 (4.8)	3,838 (6.9)	
Wealthy	7,447 (26.1)	6,301 (23.1)	13,748 (24.7)	
Average	12,709 (44.6)	13,517 (49.6)	26,226 (47.1)	
Poor	4,477 (15.7)	4,921 (18.1)	9,398 (16.9)	
Very poor	1,339 (4.7)	1,182 (4.3)	2,521 (4.5)	
**Living situation**				<0.0001
Living with family	27,133 (95.2)	26,070 (95.7)	53,203 (95.5)	
Living with relatives	319 (1.1)	283 (1.0)	602 (1.1)	
Boarding house, living alone, dormitory	963 (3.4)	841 (3.1)	1,804 (3.2)	
Facilities*	86 (0.3)	36 (0.1)	122 (0.2)	
**City type**				<0.0001
Large city	13,958 (49.0)	12,776 (46.9)	26,734 (48.0)	
Mid-sized city	10,750 (37.7)	10,953 (40.2)	21,703 (38.9)	
Small city	3,793 (13.3)	3,501 (12.9)	7,294 (13.1)	
**Stress**				<0.0001
Very high	2,872 (10.1)	4,101 (15.1)	6,973 (12.5)	
High	7,708 (27.0)	9,550 (35.1)	17,258 (31.0)	
Moderate	12,409 (43.5)	10,407 (38.2)	22,816 (40.9)	
Low	4,613 (16.2)	2,860 (10.5)	7,473 (13.4)	
None	899 (3.2)	312 (1.15)	1,211 (2.2)	
**Atopic dermatitis with severity** †				<0.0001
0	22,951 (80.5)	20,266 (74.4)	43,217 (77.6)	
1	5,317 (18.7)	6,798 (25.0)	12,115 (21.7)	
2	151 (0.5)	110 (0.4)	261 (0.5)	
3	36 (0.1)	27 (0.1)	63 (0.1)	
4	46 (0.2)	29 (0.1)	75 (0.1)	

* Facilities: an organization for social welfare and an orphanage.

† Atopic dermatitis with severity: (0) no atopic dermatitis, (1) having atopic dermatitis but no school absence, (2) 1–3 days of school absence with atopic dermatitis, (3) 4–6 days of school absence with atopic dermatitis, and (4) more than 7 days of school absence with atopic dermatitis.

### Dependent variable

A history of atopic dermatitis was assessed by asking each adolescent, “In your life, have you ever been diagnosed with atopic dermatitis by a doctor?” The responses were: (1) no and (2) yes. Also the severity of atopic dermatitis was ascertained by the question, “Have you been absent from school due to atopic dermatitis in the last twelve months?” The responses were: (1) no absence, (2) 1–3 days absence, (3) 4–6 days absence, and (4) more than 7 days absence. To consider the severity of atopic dermatitis in our analysis, we combined two variables and created an ordinal variable with five categories as (0) no atopic dermatitis, (1) having atopic dermatitis but no school absence, (2) 1–3 days of school absence with atopic dermatitis, (3) 4–6 days of school absence with atopic dermatitis, and (4) more than 7 days of school absence with atopic dermatitis.

### Independent variable of main interest

Self-reported perceived stress was assessed for each adolescent by asking, “How much stress are you experiencing in your daily life?” The responses were: (1) a very high level, (2) a high level, (3) a moderate level, (4) a low level, and (5) none.

### Covariate variables

Age: Age data were obtained from the KYRBWS-VI, and no alterations were made.

Current smoking: Current smoking status was evaluated in each participant by asking, “In the last 30 days, how many days have you smoked more than one cigarette?” The response options were: (1) none, (2) 1–2 days per month, (3) 3–5 days per month, (4) 6–9 days per month, (5) 10–19 days per month, (6) 20–29 days per month, and (7) every day. From these options, responses were grouped into two categories. Participants whose responses ranged from (2) to (7) were considered smokers, and those whose response was (1) were considered nonsmokers.

Current alcohol consumption: Current alcohol consumption was evaluated in each participant by asking, “In the last 30 days, how many days have you consumed more than one glass of alcohol?” The response options were: (1) none, (2) 1–2 days per month, (3) 3–5 days per month, (4) 6–9 days per month, (5) 10–19 days per month, (6) 20–29 days per month, and (7) every day. Participants were grouped into two categories: those whose responses ranged from (2) to (7) were considered consumers of alcohol, and those whose response was (1) were considered non-consumers.

Maternal educational level: Maternal educational level was assessed by asking, “What is your mother's educational level?” Response options were: (1) middle school or lower, (2) high school, and (3) college or higher.

Paternal educational level: Paternal educational level was assessed by asking, “What is your father's educational level?” Response options were: (1) middle school or lower, (2) high school, and (3) college or higher.

Economic status: Economic status was evaluated by asking, “What is your parents' economic status?” Response options were: (1) very wealthy, (2) wealthy, (3) average, (4) poor, and (5) very poor.

Living situation: The living situation of each participant was assessed by asking, “What is your current living situation?” The responses were: (1) living with immediate family; (2) living with relatives; (3) living in a boarding house, living alone, or living in a dormitory; and (4) living in facilities such as an organization for social welfare and an orphanage.

City type: These data were obtained from the KYRBWS-VI, and no alterations were made (large, mid-sized, and small city).

### Statistical analysis

Besides the status of atopic dermatitis, we also considered the severity of atopic dermatitis by incorporating a variable on the number of absent school days due to atopic dermatitis and using the dependent variable with five categories: (0) no atopic dermatitis, (1) having atopic dermatitis but no school absence, (2) 1–3 days of school absence with atopic dermatitis, (3) 4–6 days of school absence with atopic dermatitis, and (4) more than 7 days of school absence with atopic dermatitis. We conducted an ordinal logistic regression analysis to investigate the association between stress level and the diagnosis and severity of atopic dermatitis. First, a univariate ordinal logistic regression analysis of atopic dermatitis conditions was performed. Next, multivariable ordinal logistic regression analysis adjusted for age, current smoking status, current alcohol consumption, maternal educational level, paternal educational level, economic status, living situation, and type of city was performed. All data were represented as *N* (%), and statistical significance was set at *P*<0.05. Odds ratios and 95% confidence interval (CI) were calculated. Statistical analyses were performed using SAS, version 9.2 (SAS Institute Inc., Cary, NC, US).

## Results

A total of 5,550 boys and 6,964 girls were diagnosed with atopic dermatitis at baseline. Table 1 shows the baseline characteristics of the population.

### Univariate ordinal logistic regression analyses

Students' age was highly significantly associated with atopic dermatitis. Younger students were more likely to suffer from atopic dermatitis and to have more severe condition (Table 2). The trend of younger students having higher risk of atopic dermatitis was particularly clear among boys (compared with 18-year-old boys, the odds ratios (OR) were 1.33 (95% CI, 1.13–1.56; *P*<0.0001) for 12-year-old boys, 1.24 (95% CI, 1.10–1.40; *P*<0.0001) for 13-year-old boys, 1.15 (95% CI, 1.02–1.30; *P* = 0.02) for 14-year-old boys and that for 15-year-old boys was 1.15 (95% CI, 1.02–1.30; *P* = 0.02). Similar trend in the association between age and atopic dermatitis was shown in girls (Table 2).

**Table 2:** Factors associated with atopic dermatitis using ordinal logistic regression (univaraite analysis).

Variables	Boys			Girls			Total		
	OR	95% CI	*P*-value	OR	95% CI	*P*-value	OR	95% CI	*P*-value
**Age (years)**									
12	1.33	1.13–1.56	<.0001	1.26	1.09–1.46	<.0001	1.30	1.17–1.45	<.0001
13	1.24	1.10–1.40	<.0001	1.41	1.26–1.58	<.0001	1.32	1.22–1.44	<.0001
14	1.15	1.02–1.30	0.02	1.32	1.18–1.47	<.0001	1.24	1.14–1.34	<.0001
15	1.15	1.02–1.30	0.02	1.21	1.09–1.36	<.0001	1.18	1.09–1.28	<.0001
16	1.10	0.98–1.24	0.11	1.12	1.00–1.25	0.05	1.11	1.03–1.21	0.01
17	1.11	0.98–1.25	0.09	1.07	0.96–1.20	0.21	1.09	1.01–1.19	0.03
18	1.00			1.00			1.00		
**Current smoking**									
Nonsmoking	1.00			1.00			1.00		
Smoking	0.90	0.83–0.98	0.01	0.99	0.88–1.11	0.83	0.86	0.80–0.91	<.0001
**Current alcohol consumption**									
Nondrinking	1.00			1.00			1.00		
Drinking	0.96	0.89–1.02	0.20	0.96	0.89–1.03	0.20	0.93	0.89–0.98	<.0001
**Educational level of mother**									
Middle school or lower	1.00			1.00			1.00		
High school	1.15	1.01–1.30	0.03	1.37	1.21–1.54	<.0001	1.26	1.16–1.38	<.0001
College or higher	1.33	1.17–1.51	<.0001	1.59	1.40–1.79	<.0001	1.45	1.32–1.58	<.0001
**Educational level of father**									
Middle school or lower	1.00			1.00			1.00		
High school	1.12	0.99–1.28	0.08	1.28	1.13–1.44	<.0001	1.21	1.11–1.32	<.0001
College or higher	1.41	1.24–1.60	<.0001	1.45	1.29–1.64	<.0001	1.43	1.31–1.56	<.0001
**Economic status**									
Very wealthy	0.93	0.79–1.10	0.41	1.00	0.83–1.19	0.97	0.92	0.81–1.03	0.15
Wealthy	1.04	0.90–1.20	0.60	1.07	0.93–1.23	0.35	1.05	0.95–1.16	0.34
Average	0.93	0.81–1.08	0.34	0.94	0.82–1.07	0.36	0.95	0.86–1.05	0.31
Poor	0.95	0.82–1.11	0.54	0.94	0.82–1.09	0.44	0.97	0.87–1.07	0.50
Very poor	1.00			1.00			1.00		
**Living situation**									
Living with family	0.86	0.52–1.43	0.55	1.33	0.59–2.98	0.49	1.05	0.68–1.62	0.82
Living with relatives	0.95	0.54–1.69	0.86	1.51	0.65–3.53	0.34	1.17	0.74–1.88	0.50
Boarding house, living alone, dormitory	0.78	0.45–1.33	0.35	1.28	0.56–2.92	0.55	0.98	0.63–1.52	0.92
Facilities*	1.00			1.00			1.00		
**City type**									
Large city	1.22	1.11–1.35	<.0001	1.01	0.93–1.10	0.80	1.10	1.04–1.18	<.0001
Mid-sized city	1.18	1.07–1.31	<.0001	1.06	0.97–1.15	0.23	1.12	1.05–1.20	<.0001
Small city	1.00			1.00			1.00		
**Stress**									
Very high	1.41	1.16–1.72	<.0001	1.15	0.88–1.50	0.32	1.46	1.25–1.70	<.0001
High	1.40	1.16–1.68	<.0001	1.14	0.88–1.48	0.33	1.43	1.23–1.66	<.0001
Moderate	1.18	0.98–1.41	0.08	1.03	0.79–1.34	0.82	1.22	1.05–1.41	0.01
Low	1.10	0.90–1.33	0.35	0.90	0.69–1.19	0.47	1.07	0.92–1.26	0.37
None	1.00			1.00			1.00		

* Facilities: an organization for social welfare and an orphanage.

The association between current smoking status and atopic dermatitis in boys was statistically significant with an odds ratio of 0.90 (95% CI, 0.83–0.98; *P* = 0.01). When boys and girls were combined, the odds ratio was 0.86 (95% CI, 0.80–0.91; *P*<0.0001) for smoking compared with nonsmoking. The odds ratio between current alcohol consumption and atopic dermatitis in total was 0.93 (95% CI, 0.89–0.98; *P*<0.0001, Table 2).

In terms of the relationship between educational level of mother and atopic dermatitis in boys, the odds ratio was 1.15 (95% CI, 1.01–1.30; *P* = 0.03) for those whose mothers had attended high school, and 1.33 (95% CI, 1.17–1.51; *P*<0.0001) for those with mothers whose education was college or higher compared with those whose mothers was middle school or lower. The odds ratios in girls were 1.37 (95% CI, 1.21–1.54; *P*<0.0001) for those whose mothers had attended high school and 1.59 (95% CI, 1.40–1.79; *P*<0.0001) for those whose mothers had attended at least college compared with those whose mothers had finished middle school at most. In total, the odds ratios for educational level of mother and atopic dermatitis were 1.26 (95% CI, 1.16–1.38; *P*<0.0001) for those with mothers with a high school education and 1.45 (95% CI, 1.32–1.58; *P*<0.0001) for those with mothers with a college or higher education compared with those whose mothers had attended middle school at most (Table 2).

With respect to the association between educational level of father and atopic dermatitis in boys, the odds ratio was 1.41 (95% CI, 1.24–1.60; *P*<0.0001) for those whose fathers attended to college or higher compared with those whose fathers attended middle school at most. The odds ratio in girls was 1.28 (95% CI, 1.13–1.44; *P*<0.0001) for those with fathers with a high school education and 1.45 (95% CI, 1.29–1.64; *P*<0.0001) for those with father whose education included college or more compared with those with father whose education included middle school at most. In total, the odds ratios for educational level of father and atopic dermatitis were 1.21 (95% CI, 1.11–1.32; *P*<0.0001) for those whose fathers attended high school and 1.43 (95% CI, 1.31–1.56; *P*<0.0001) for those whose fathers attended college or higher compared with those whose fathers attended middle school at most (Table 2).

The odds ratios for type of city type and atopic dermatitis in boys were 1.22 (95% CI, 1.11–1.35; *P*<0.0001) for those living in a large city and 1.18 (95% CI, 1.07–1.31; *P*<0.0001) for those living in a mid-sized city compared with those living in a small city. However, the relationship between city type and atopic dermatitis was not statistically significant among girls. In total, the odds ratios were 1.10 (95% CI, 1.04–1.18; *P*<0.0001) for adolescents living in large cities and 1.12 (95% CI, 1.05–1.20; *P*<0.0001) for those living in mid-sized cities compared with those living in small cities (Table 2).

Among boys, the odds ratios for stress and diagnosis with atopic dermatitis were 1.41 (95% CI, 1.16–1.72; *P*<0.0001) for very high stress, 1.40 (95% CI, 1.16–1.68; *P*<0.0001) for high stress compared with no stress. However, we found no statistically significant relationship between stress and diagnosis with atopic dermatitis in girls. In total, the odds ratios for stress and diagnosis with atopic dermatitis were 1.46 (95% CI, 1.25–1.70; *P*<0.0001) for those with very high levels of stress, 1.43 (95% CI, 1.23–1.66; *P*<0.0001) for those with high levels of stress, and 1.22 (95% CI, 1.05–1.41; *P* = 0.01) for those with moderate levels of stress compared with those with no stress (Table 2).

### Multivariable ordinal logistic regression analyses

The results of the multivariable ordinal logistic regression analyses of stress and atopic dermatitis are presented in Table 3. The results were adjusted for age, current smoking status, current alcohol consumption, educational level of mother, educational level of father, economic status, living situation, and whether students resided in large, medium, or small cities. The value of stress level, the independent variable, ranged from (1) very high to (5) none.

**Table 3:** Factors associated with atopic dermatitis using ordinal logistic regression (multivariable analysis).

Variables	Boys			Girls			Total		
	OR	95% CI	*P*-value	OR	95% CI	*P*-value	OR	95% CI	*P*-value
**Age (years)**									
12	1.33	1.13–1.56	<.0001	1.25	1.08–1.46	<.0001	1.30	1.17–1.45	<.0001
13	1.24	1.09–1.40	<.0001	1.41	1.26–1.58	<.0001	1.33	1.22–1.44	<.0001
14	1.15	1.02–1.30	0.02	1.32	1.18–1.48	<.0001	1.24	1.14–1.35	<.0001
15	1.16	1.03–1.31	0.02	1.22	1.09–1.37	<.0001	1.19	1.10–1.29	<.0001
16	1.12	1.00–1.26	0.06	1.12	1.00–1.25	0.05	1.13	1.04–1.22	<.0001
17	1.12	1.00–1.27	0.06	1.08	0.97–1.21	0.17	1.10	1.02–1.20	0.02
18	1.00			1.00			1.00		
**Current smoking**									
Nonsmoking	1.00			1.00			1.00		
Smoking	0.93	0.85–1.01	0.10	1.02	0.90–1.15	0.76	0.89	0.83–0.95	<.0001
**Current alcohol consumption**									
Nondrinking	1.00			1.00			1.00		
Drinking	1.02	0.94–1.10	0.68	0.98	0.91–1.06	0.68	1.00	0.94–1.05	0.88
**Educational level of mother**									
Middle school or lower	1.00			1.00			1.00		
High school	1.05	0.91–1.20	0.52	1.28	1.12–1.46	<.0001	1.16	1.06–1.28	<.0001
College or higher	1.07	0.92–1.25	0.36	1.41	1.22–1.63	<.0001	1.23	1.11–1.36	<.0001
**Educational level of father**									
Middle school or lower	1.00			1.00			1.00		
High school	1.10	0.96–1.26	0.19	1.15	1.01–1.32	0.04	1.14	1.03–1.25	0.01
College or higher	1.34	1.15–1.54	<.0001	1.22	1.06–1.41	<.0001	1.29	1.16–1.42	<.0001
**Economic status**									
Very wealthy	0.86	0.72–1.02	0.08	0.86	0.71–1.03	0.10	0.83	0.73–0.94	<.0001
Wealthy	0.98	0.84–1.14	0.75	0.94	0.81–1.09	0.44	0.96	0.86–1.07	0.44
Average	0.92	0.80–1.06	0.26	0.87	0.76–1.00	0.05	0.91	0.82–1.01	0.06
Poor	0.96	0.82–1.12	0.60	0.91	0.79–1.06	0.23	0.95	0.85–1.06	0.33
Very poor	1.00			1.00			1.00		
**Living situation**									
Living with family	0.84	0.50–1.41	0.50	1.35	0.60–3.06	0.47	1.01	0.65–1.57	0.96
Living with relatives	0.94	0.53–1.69	0.84	1.56	0.66–3.67	0.31	1.15	0.72–1.85	0.55
Boarding house, living alone, dormitory	0.79	0.46–1.37	0.40	1.37	0.60–3.14	0.46	0.98	0.63–1.55	0.95
Facilities*	1.00			1.00			1.00		
**City type**									
Large city	1.15	1.05–1.27	<.0001	0.97	0.88–1.06	0.44	1.05	0.98–1.12	0.18
Mid-sized city	1.12	1.02–1.24	0.02	1.02	0.93–1.11	0.68	1.07	1.00–1.14	0.04
Small city	1.00			1.00			1.00		
**Stress**									
Very high	1.46	1.20–1.78	<.0001	1.24	0.94–1.62	0.12	1.53	1.31–1.78	<.0001
High	1.44	1.19–1.73	<.0001	1.22	0.94–1.59	0.14	1.48	1.27–1.72	<.0001
Moderate	1.21	1.00–1.45	0.05	1.09	0.84–1.41	0.54	1.24	1.07–1.45	<.0001
Low	1.10	0.91–1.33	0.34	0.93	0.71–1.22	0.59	1.08	0.92–1.26	0.36
None	1.00			1.00			1.00		

* Facilities: an organization for social welfare and an orphanage.

Similar to the results of univariate analysis, age was very significantly associated with atopic dermatitis and the severity of condition. Compared to 18-year-old boys, the odds ratios of having atopic dermatitis with severity were 1.33 (95% CI, 1.13–1.56; *P*<0.0001) for 12-year-old boys, 1.24 (95% CI, 1.09–1.40; *P*<0.0001) for 13-year-old boys, 1.15 (95% CI, 1.02–1.30; *P* = 0.02) for 14-year-old boys, 1.16 (95% CI, 1.03–1.31; *P* = 0.02) for 15-year-old boys, 1.12 (95% CI, 1.00–1.26; *P* = 0.06) for 16-year-old boys, and 1.12 (95% CI, 1.00–1.27; *P* = 0.06) for 17-year-old boys compared with boys aged 18 years (Table 3). Similar trend was observed in girls. Compared with 18-year-old girls the odds ratios were 1.25 (95% CI, 1.08–1.46; *P*<0.0001) for 12-year-old girls and 1.41 (95% CI, 1.26–1.58; *P*<0.0001) for 13-year-old girls (Table 3). In total, the odds ratios were 1.30 (95% CI, 1.17–1.45; *P*<0.0001) for 12-year-old, 1.33 (95% CI, 1.22–1.44; *P*<0.0001) for 13-year-old, 1.24 (95% CI, 1.14–1.35; *P*<0.0001) for 14-year-old, 1.19 (95% CI, 1.10–1.29; *P*<0.0001) for 15-year-old, 1.13 (95% CI, 1.04–1.22; *P*<0.0001) for 16-year-old, and 1.10 (95% CI, 1.02–1.20; *P* = 0.02) for 17-year-old compared with aged 18 years (Table 3). The odds ratio for current smokers compared to nonsmokers was 0.89 (95% CI, 0.83–0.95; *P*<0.0001). Alcohol drinking was not significantly associated with atopic dermatitis (Table 3).

With respect to the educational level of mother, the odds ratios for atopic dermatitis in girls were 1.28 (95% CI, 1.12–1.46; *P*<0.0001) for those with mothers who had attended high school and 1.41 (95% CI, 1.22–1.63; *P*<0.0001) for those whose mothers had attended college or higher compared with those whose mothers had attended middle school at most. In total, the odds ratios were 1.16 (95% CI, 1.06–1.28; *P*<0.0001) for those with mothers who had attended high school and 1.23 (95% CI, 1.11–1.36; *P*<0.0001) for those whose mothers had attended college or higher compared with those whose mothers had attended middle school at most (Table 3).

With respect to educational level of father, the odds ratio for atopic dermatitis in boys whose fathers attended college or higher was 1.34 (95% CI, 1.15–1.54; *P*<0.0001) compared with those whose fathers attended middle school at most. In girls, the odds ratios whose fathers attended high school was 1.15 (95% CI, 1.01–1.32; *P* = 0.04), and whose fathers attended college or higher was 1.22 (95% CI, 1.06–1.41; *P*<0.0001). In total, the odds ratio for atopic dermatitis in adolescents whose fathers attended high school was 1.14 (95% CI, 1.03–1.25; *P* = 0.01), and whose fathers attended college or higher was 1.29 (95% CI, 1.16–1.42; *P*<0.0001) compared with those whose fathers attended middle school at most (Table 3).

With respect to size of city, the odds ratio for atopic dermatitis in boys in a large city was 1.15 (95% CI, 1.05–1.27 *P*<0.0001), and in a mid-sized city was 1.12 (95% CI, 1.02–1.24 *P* = 0.02), compared with those living a small city. In total, the odds ratio was 1.07 (95% CI, 1.00–1.14; *P* = 0.04) for adolescents in a mid-sizes city compared with those living in a small city (Table 3).

The odds ratios for stress and atopic dermatitis in boys with very high stress levels were 1.46 (95% CI, 1.20–1.78; *P*<0.0001), 1.44 for those with high stress levels (95% CI, 1.19–1.73; *P*<0.0001), and 1.21 for those with moderate stress levels (95% CI, 1.00–1.45; *P* = 0.05) compared with those no stress. In contrast, we found no statistically significant relationship between stress and atopic dermatitis in girls. In total, the odds ratios for stress and atopic dermatitis were 1.53 (95% CI, 1.31–1.78; *P*<0.0001) for those with very high stress, 1.48 (1.27–1.72, 95% CI; *P*<0.0001) for those with high stress, and 1.24 (95% CI, 1.07–1.45; *P*<0.0001) for those with moderate stress compared with those with no stress. The *P*-value for the trend analysis of stress, *P*<0.0001, was statistically significant. The association between stress and atopic dermatitis increased as a function of increasing stress (Table 3).

## Discussion

Adolescence is accompanied by rapid and varied changes in physical growth, sexual maturity, hormone levels, and psychological issues [33], [34]. Moreover, this population may not have the maturity to overcome stressful situations. Therefore, adolescents are at risk for conditions associated with stress. This study investigated the relationship between level of stress and atopic dermatitis in Korean adolescents.

In this study, we analyzed the relationship between stress level and atopic dermatitis, adjusting for age, current smoking status, current alcohol consumption, maternal educational level, paternal educational level, economic status, living situation, and size of city. Besides, atopic dermatitis' severity was also considered by including the information on the number of absent school days due to atopic dermatitis. Our data showed that girls aged 13 and 14 years of age had particularly higher risk of having more severe atopic dermatitis and this may be due to the changes in hormones associated with menarche. According to the KYRBWS-VI survey, the mean age of menarche was 12 years old. At the beginning of menstruation, hormonal changes may play a significant role in increasing atopic dermatitis' severity during puberty and adolescence. According to Osman et al., female sex hormones released during the menstrual cycle affect the expression of atopic dermatitis [35]. Moreover, 30–50% of women in the premenstrual phase experience an exacerbation of atopic dermatitis symptoms, and the ovulatory phase aggravates the condition of atopic dermatitis [36]. In addition to skin reactivity, bronchial hyper-responsiveness increases as a function of the menstrual cycle and peaks during the luteal and follicular phases [37].

Our study results show that the risk of having atopic dermatitis and severe condition increased with higher maternal educational levels in girls and with higher paternal educational levels in both boys and girls. Boys and girls with highly educated parents were more likely to have atopic dermatitis with increased severity than were boys and girls with less educated parents. Indeed, parents who are highly educated are more likely to recognize the symptoms of atopic dermatitis and to see doctors than are parents with less education [38]. Additionally, boys who resided in large cities and mid-sizes cities were more likely to report having been diagnosed with severe atopic dermatitis than were those living in small cities. This result implies that big cities may have more sources of pollution that stimulate the autoimmune system [39]. Also, the results from educational level and city type showed that students having parents with high educational level and living in large cities were more likely to have atopic dermatitis. According to Xu et al., there was a significant correlation between among prevalence of atopic dermatitis, GDP (Gross Domestic Product), and the city size. The prevalence of atopic dermatitis was higher in the communities with higher GDP and larger city size. [40]. Also, Shaw et al. reported that atopic dermatitis prevalence was affected by high education level, living in metropolitan area, and high socioeconomic status [41]. In Korea, according to Lee et al., as monthly income and education status increase, there was more severe atopic dermatitis populations observed [42]. Also, Werner et al. and Snijders et al. reported the relationship between a privileged life-style and an increased incidence of AD [43], [44]. Therefore, there are relationship among socioeconomic status, educational level, and city size with prevalence of atopic dermatitis.

The results of this study show that probability of developing atopic dermatitis was positively associated with increasing stress levels (very high, high, and moderate compared with none) only in boys. One explanation for this result may involve sex-related differences in stress-related HPA axis reactions to psychological stressors [29]. The cortisol responses of young men are more pronounced than are those of young women after psychological stress [29]. Additionally, the *P*-values for the trend analysis reflected a positive association with atopic dermatitis. Parents or care providers who treat adolescents with atopic dermatitis should consider not only whether a child was stressed but also the degree of stress.

From the Sixth KYRBWS-VI data in 2010 in Korea, smoking rate of aged 12–18 was 16.6% in boys and 7.1% in girls [30]. In our data, girls' smoking prevalence was lower than that of boys and this trend was also observed in another Korean national data [45]. The discrepancy in smoking prevalence between boys and girls may be explained by the social norms influenced by Korean Confusion culture that smoking women are not culturally well-accepted in Korea. Compared to other countries in Organisation for Economic Co-operation and Development (OECD), Korean boys had relatively lower smoking rates: Turkey (14.4%), United States of America (15.4%), Swaziland (15.8%), New Zealand (18.7%), Poland (26.0%), Mexico (27.8%), Hungary (27.9%), Slovakia (28.5%), Estonia (33.8%), and Czech Republic (35.8%), respectively. Smoking rates of Korean girls were also lower than other OECD countries: Turkey (7.4%), Swaziland (8.6%), United States of America (11.1%), New Zealand (21.5%), Slovakia (24.5%), Hungary (26.7%), Estonia (27.8%), Mexico (28.5%), Poland (31.7%), and Czech Republic (34.1%) [45]. These data indicated that Korean adolescents' smoking rate was relatively low among OECD countries.

According to the KYRBWS-VI data in 2010 in Korea, the prevalence of alcohol consumption of 7th to the 12th grades was 23.5% in boys and 18.3% in girls [30]. Based on the national Youth Risk Behavior Survey monitors in the US in 2011, the alcohol consumption rate of 9th through 12th grade students of boys was 39.5%, and girls was 37.9% [46]. These data showed that Korean adolescents' alcohol consumption rate was lower than the US.

To our knowledge, this is the first study to examine the association between stress and severity of atopic dermatitis in an Asian population using national survey data. The survey was large in scale and relied on a complex design including multistage sampling, stratification, and clustering. The reliability and validity of the questionnaire data were assessed by previous studies [31], [32]. Additionally, the response rate to the survey was 97.7% (73,238 of a possible total of 74,980), representing a majority of Korean adolescents.

This study has several limitations. Using data from the KYRBWS-VI, this study was cross-sectional in design; thus, it examined the interrelationship between stress and atopic dermatitis rather than issues of causality. Additionally, some confounding variables may not have been included in this study. Data on the socioeconomic status of the respondents may not be accurate because students rather than parents provided this information. However, according to Chang's study, the survey result from 442 participants in Korea showed that there was a significant correlation between education and income [47]. It seems that higher education provides more opportunities to get a better job and monetary benefit compare to lower education in Korea. Therefore, the educational level of parents could be used as a proxy variable of socioeconomic status in Korea.

As we relied on self-reports of stress level, there may be some limitation in the assessment of each student's stress level. However, the questions we used for self-reported stress level are standard, and similar questions have been used in other studies such as a Canadian nationwide health survey where self-perceived stress level was assessed by choosing among the five categories: (1) not at all, (2) not very stressful, (3) a bit, (4) quite a bit, and (5) extremely stressful [48]. The survey asked about whether each adolescent had ever been diagnosed with atopic dermatitis and absent school days due to atopic dermatitis, but it did not gather information regarding time of diagnosis. Therefore, the results from this study should be interpreted carefully. Future studies should investigate time of diagnosis with atopic dermatitis to clarify the causal relationship between stress and atopic dermatitis.
